# Chemical and Genetic Validation of the Statin Drug Target to Treat the Helminth Disease, Schistosomiasis

**DOI:** 10.1371/journal.pone.0087594

**Published:** 2014-01-29

**Authors:** Liliana Rojo-Arreola, Thavy Long, Dan Asarnow, Brian M. Suzuki, Rahul Singh, Conor R. Caffrey

**Affiliations:** 1 Center for Discovery and Innovation in Parasitic Diseases, Department of Pathology, University of California San Francisco, San Francisco, California, United States of America; 2 Department of Computer Science, San Francisco State University, San Francisco, California, United States of America; Swiss Tropical and Public Health Institute, Switzerland

## Abstract

The mevalonate pathway is essential in eukaryotes and responsible for a diversity of fundamental synthetic activities. 3-hydroxy-3-methylglutaryl coenzyme A reductase (HMGR) is the rate-limiting enzyme in the pathway and is targeted by the ubiquitous statin drugs to treat hypercholesterolemia. Independent reports have indicated the cidal effects of statins against the flatworm parasite, *S. mansoni*, and the possibility that SmHMGR is a useful drug target to develop new statin-based anti-schistosome therapies. For six commercially available statins, we demonstrate concentration- and time-dependent killing of immature (somule) and adult *S. mansoni in vitro* at sub-micromolar and micromolar concentrations, respectively. Cidal activity trends with statin lipophilicity whereby simvastatin and pravastatin are the most and least active, respectively. Worm death is preventable by excess mevalonate, the product of HMGR. Statin activity against somules was quantified both manually and automatically using a new, machine learning-based automated algorithm with congruent results. In addition, to chemical targeting, RNA interference (RNAi) of HMGR also kills somules *in vitro* and, again, lethality is blocked by excess mevalonate. Further, RNAi of HMGR of somules *in vitro* subsequently limits parasite survival in a mouse model of infection by up to 80%. Parasite death, either via statins or specific RNAi of HMGR, is associated with activation of apoptotic caspase activity. Together, our genetic and chemical data confirm that *S. mansoni* HMGR is an essential gene and the relevant target of statin drugs. We discuss our findings in context of a potential drug development program and the desired product profile for a new schistosomiasis drug.

## Introduction

The mevalonate pathway is essential in eukaryotes [1], [2]. Branches arising from the central pathway are responsible for a diversity of fundamental synthetic activities, e.g., sterols, dolichol and ubiquinone, and for protein prenylation and glycosylation [3]–[5]. The enzyme responsible for the rate-limiting catalytic step in the pathway, 3-hydroxy-3-methylglutaryl coenzyme A (HMG-CoA) reductase (HMGR), is a trans-membrane glycoprotein in the endoplasmic reticulum that is responsible for converting HMG-CoA to mevalonate [6]. Specific inhibitors of this enzyme, termed statins, were first identified in the early 1970's from *Penicillium* and *Aspergillus* fungi [7]. Their ability to competitively inhibit cholesterol synthesis led to the development of the now ubiquitous anti-hypercholesterolemia drugs to control cardiovascular disease [8], [9]. Statins have been ‘blockbuster’ drugs for the major pharmaceutical companies with sales in excess of $12 billion dollars per year between 2005 and 2008 [10].

In addition to hypercholesterolemia, other potential therapeutic uses have emerged for statins, including the treatment of inflammatory [11] and immune disorders [12], and as anti-cancer drugs [13], [14]. Statins have also been explored as possible treatments for parasitic diseases, including Human African Trypanosomiasis [15], Chagas' disease [16], leishmaniasis [17] and malaria [18]–[20].

HMGR has been described and characterized in the flatworm parasite, *Schistosoma mansoni* [21], [22], which is one of a number of schistosome species that cause the globally prevalent disease, schistosomiasis [23], [24]. Whereas in mammals, HMGR homeostasis is regulated both transcriptionally and post-transcriptionally by cholesterol levels and conformational changes induced by phosphorylation state [25]–[28], the regulation of biosynthesis and activity of the *S. mansoni* HMGR (SmHMGR) are unknown. The presence of sterols and non-sterol lipids in the diet of the host does not affect parasite enzyme activity and there is no evidence of a reductase phosphatase-kinase system, therefore, it is presumed that the mechanism differs from that described in mammals [29]. Also, in schistosomes, the branch responsible for *de novo* synthesis of cholesterol is absent – a feature that is shared among worms [2], [30], [31].

The HMGR statin inhibitor, lovastatin, severely impacted egg-production [32], [33] and survival of *S. mansoni* in a mouse model of infection [34]. Parasite death could be prevented in the presence of excess mevalonate, the specific product of HMGR [34]. Subsequently, our own observations confirmed the lethality of statins to *S. mansoni in vitro* during a phenotypic (whole organism) screening campaign employing drugs approved for use in humans [35]. These data support the continued investigation of SmHMGR as a possible drug target, especially considering that just one drug, praziquantel (PZQ), is available to treat and control the disease [24], [36]. Also, most of the erstwhile blockbuster statins are now off-patent relaxing intellectual property constraints for further chemical development.

To follow up on these observations and in the further consideration of SmHMGR as a possible drug target, we measured the *in vitro* bioactivities of six commercially available statins against both the somule (post-infective larvae) and adult stages of *S. mansoni*. For somules, we also developed an automated screening strategy to measure statin bioactivity. In addition to the chemical approach using statins, we also employed the reverse genetics tool of RNA interference (RNAi) to understand the contribution of SmHMGR to somule survival both *in vitro* and in an ‘*in vitro*-to-*in vivo*’ method, whereby parasites are first subjected to RNAi *in vitro* before transfer into a mouse model of infection. Finally, we attempt to identify by which mechanism(s) interference with SmHMGR function leads to parasite death. The results are discussed with respect to the current target product profile for schistosomiasis, not least the goal of achieving single oral dosing, which is the case with the current drug, PZQ.

## Materials and Methods

### Ethics statement

Maintenance and handling of small mammals were carried out in accordance with a protocol (AN086607-03) approved by the Institutional Animal Care and Use Committee (IACUC) of the University of California San Francisco.

### *Schistosoma mansoni* life cycle and parasite maintenance *in vitro*

The acquisition, preparation and *in vitro* maintenance of newly transformed schistosomula (somules; derived from infective stage cercariae) and 42-day-old adult *S. mansoni* (from small mammals) have been described by us previously [35], [37].

### Chemical screens of somules and adult *S. mansoni* with commercial statins

Six commercially available statins, namely, atorvastatin, fluvastatin, lovastatin, pravastatin, rosuvastatin and simvastatin (available from Sigma-Aldrich, Tocris or LC Laboratories) were dissolved as 20 mM stock solutions in DMSO. For *in vitro* tests with somules and adults, serial dilutions were set up as indicated in the results section. Final concentrations of DMSO were never greater than 0.5%. Somules were distributed in 96-well flat-bottomed plates at a density of 300 worms in 200 µL culture medium. Culture medium was Basch medium 169 [38] (custom prepared at the UCSF Cell Culture Facility) containing 5% FBS, 100 U/ml penicillin and 100 mg/ml streptomycin solution. Plates were kept at 37°C and 5% CO_2_. For screens with adult *S. mansoni*, five pairs were maintained in 2 mL of the above medium in 24-well culture plates. To assess whether mevalonate, the product of HMGR, could rescue any deleterious phenotype induced by statins, the compound was added at the final concentrations indicated in the results section from a stock solution of 3.87 M (±)-mevalonolactone (Sigma-Aldrich) dissolved in water.

Parasite phenotypic responses to statins were recorded daily out to four and seven days for somules and adults, respectively. For both developmental stages, the process of manually adjudicating the manifold phenotypic responses possible by this parasite has been described [35] and essentially involves recording changes in movement, shape, translucence, surface integrity and, in the case of adults, the ability of the parasite to adhere to the culture dish surface. With somules, those statins that were bioactive consistently induced a darkening and rounding of the parasite and impaired motility in a process we termed ‘degeneration.’ Each day representative photographic images were taken and the number of degenerate parasites calculated as a percentage of the total number of worms in the given image. Images from each treatment were captured using a Zeiss Axiovert 40 C inverted microscope (10x objective) and a Zeiss AxioCam MRc digital camera controlled by AxioVision 40 (version 4.8.1.0) software.

For adults, we converted the types and number of phenotypic responses recorded manually into a ‘severity score’ ranging from 0 (no effect) to 4 (severely compromised). Each response was awarded a score of 1, except where damage to the parasite's tegument (surface) was evident, in which case the maximum score of 4 was awarded on the assumption that such damage is lethal to adult parasites, including in the mammalian host [39].

### Automated high-throughput approach to screening somules

The automatic approach for identifying and counting ‘degenerate’ and ‘normal’ somules consisted of three main stages. First, an image processing algorithm [40], developed specifically for segmentation of somules in bright-field photographs, was used to identify individual somules and subsequently extract a quantitative, feature-space representation of each parasite. This segmentation method is generic in that it can be used with any bright-field imaging device and does not rely on any *a priori* model of parasite shape. Consequently, it is highly suited to screening complex macroparasites due to their natural irregularity of shape and size as well as the unpredictability of phenotypic effects caused by drugs on the parasites. Furthermore, the segmentation method is highly accurate: in evaluations reported [40], it was found to identify 95.9% of individual schistosomula and to detect the location of their boundaries, i.e., the outer edge of the tegument, with an average accuracy of 1.3 pixels on a data set of 6,960 hand-traced schistosomula imaged under varying conditions. Owing to the accuracy of the method, a predominant majority of touching parasites were separated during segmentation. Consequently, there was no need to discard such parasites using a predefined area threshold as was done in [41].

Following segmentation, in the second stage, each parasite was represented by a vector of descriptive measurements capturing appearance, shape and texture. These features, expanded on those used previously [41]–[43], included the statistical moments of image intensities, invariant image moments [44] and two multi-scale texture measures based on either gray-level co-occurrence matrices [45] or wavelet decomposition [46].

In the final stage, vector space representation of each somule was taken as input to a machine learning algorithm which performed the classification of parasites paralleling the role played by the human expert in the manual assay. Specifically, each feature vector was used to classify the corresponding parasite as “normal” or “degenerate” using supervised machine learning with support vector machines (SVM). The SVM is a linear method which finds a hyperplane within the feature space that separates the two classes with the maximum margin, or distance between the decision surface and the closest points [47], [48]. In the training step of the SVM, a feature space hyperplane suitable for dividing degenerate and normal parasites was constructed using a set of 3,907 segmented parasites in 118 photographs (see above). These somules were manually labeled as either normal or degenerate using a custom, web-based user interface. To ensure consistent manual decision making, for each micrograph where the parasites had to be labeled, the corresponding control micrograph was displayed. The trained SVM was then applied to a separate data set composed of 23,867 individual somules taken from 438 micrographs.

We found it is necessary to model the variation between different populations of somules, captured by individual control experiments conducted simultaneously with particular drug response experiments. This was done using a two-step classification procedure in which initial SVM classification results were used to compile ‘normal’ control parasites, which were used to determine a control medoid for each group of experiments. Final class assignments were obtained from a second classification tier in which the control mediods were used as a baseline for their particular drug-response experiments.

### Synthesis of double-stranded (ds)RNA and treatment of somules with dsRNA *in vitro*

PCR primers were designed to respectively amplify N-terminal and C-terminal segments (positions 302 to 1349 and 1854 to 2626, respectively) of the open reading frame (ORF) of *S. mansoni* HMGR (GenBank M27294) using adult mixed-sex complementary (c)DNA as template. After sequence confirmation of the amplified DNA, dsRNA templates for the N-terminal and C-terminal segments were obtained by PCR amplification with the same primers now flanked with a T7 promoter sequence [37]. These amplicons were employed as templates for reverse transcription using the T7 RiboMAX express RNAi system (Promega). DsRNA integrity was assessed by 1% agarose gel electrophoresis and its concentration measured in a Nanodrop ND-1000 Spectrophotometer (Nanodrop Technologies). DsRNA to *Discosoma sp.* mCherry fluorescent protein, a protein not present in *S. mansoni*, was also prepared for use as a control.

Incubations of somules with dsRNA were set up in duplicate in 24-well culture plates at a density of 400 worms in 1 mL culture medium at 37°C and 5% CO_2_. Fifteen µg/mL of each of the dsRNAs to the N-terminal and C-terminal sections of the SmHMGR ORF or 30 µg/mL mCherry dsRNA were employed and medium and dsRNA were exchanged every three days. Incubations were continued for either 7 days for quantitative (q)RT-PCR analysis or 25 days to monitor for gross phenotypic responses of the parasite. For qRT-PCR, quantification of *S. mansoni* cystatin (AY334553.1) and cathepsin B1.1 (AJ506157) mRNA was also performed to identify possible RNAi off-targeting by the SmHMGR dsRNA. To assess whether mevalonate could prevent the appearance phenotypic responses induced by RNAi of HMGR, the compound was added each time the medium was exchanged at the final concentrations indicated in the results section. Parasite phenotypic responses to RNAi were monitored every day and when apparent we employed the same photographic imaging and manual counting strategy used to measure statin insult as described above.

### Treatment of somules with dsRNA *in vitro* and assessment of survival after transfer into mice

Incubations of somules with dsRNA were set up as six replicates in 24-well culture plates at a density of 500 worms in 1 mL Incomplete Basch medium 169 (i.e., omitting the FBS). DsRNA was added twice daily for 2.5 days each time at a concentration of 60 µg/mL (30 µg/mL of each of the dsRNAs targeting the N-terminal and C-terminal HMGR ORF sections). Parasites were then washed twice in the same medium and the somules from one of the replicate incubations assessed for transcript suppression by qRT-PCR. The parasites from each of the remaining five replicate incubations were injected sub-cutaneously using 25 gauge needles into 4–6 week-old Swiss Webster mice. Thirty-four days post-infection, the mice were euthanized and the portal system perfused: worms were counted and sexed [49].

### Isolation of RNA and cDNA, and qRT-PCR

Procedures for isolation of parasite RNA, reverse transcription to cDNA, primer design and amplification efficiency, and qPCR analysis have been detailed previously [37]. Briefly, parasite material was washed three times in PBS, transferred to Trizol reagent (Life Technologies), flash frozen in liquid nitrogen and stored at −80°C. Total RNA was isolated using the Trizol method up until phase separation in chloroform. The RNA in the aqueous phase was further purified using the RNeasy Mini Kit (Qiagen) according to the supplier instructions. The concentration of RNA was determined using a Nanodrop ND-1000 Spectrophotometer. Single-stranded cDNA was synthesized from total RNA using SuperScript III reverse transcriptase (Invitrogen) and an oligo d(T)_18_ reverse primer according to the manufacturer's protocol. The resulting cDNA was stored in water at −20°C.

Primers for gene expression analysis were designed with the Primer 3 software (http://frodo.wi.mit.edu [50]) using the parameters described previously [37]. A BLAST analysis was run to confirm the specificity of each primer. Primers were purchased from Elim Biopharm at the 200 µM scale without further purification. The PCR amplicon generated by each primer pair was visually inspected by 1% agarose gel electrophoresis to ensure the presence and correct size of a single PCR product. The amplification efficiency of each primer pair was evaluated as described [37], [51] and only those primers generating a single dissociation peak were employed. Table S1 lists the primers used for dsRNA synthesis and qPCR analysis.

QPCR was carried out as described [37] using the LightCycler 480 SYBR Green I Master mix (Roche Diagnostics) in an MX 3005P Real-Time PCR cycler (Stratagene). *S. mansoni* cytochrome C oxidase I (GenBank accession number, AF216698) served as the sample-normalizing gene transcript [52], [53]. Controls for genomic DNA contamination (minus reverse transcriptase) and reagent purity (water control) were included for each sample. Duplicate reactions were carried out in 96-well plates for each cDNA sample in a final volume of 20 µL (Stratagene). Using the MxPro qPCR Software version 4.01 (Stratagene), the 2^−ΔΔCT^ method [54] was employed to compare transcript levels following dsRNA treatments. Transcript levels were expressed as a percentage relative to those after exposure to mCherry dsRNA.

### Measuring caspase activities after incubation with statins or HMGR dsRNA

Somules were exposed to statins or dsRNA as described above and then assessed for caspase activity as described [55]. Parasites were washed once in PBS and mixed with 100 µL lysis buffer (50 mM HEPES, pH 7.4, 100 mM NaCl, 0.5% NP-40, 1 mM EDTA, 1 mM EGTA, 1% Triton X-100 and 1% deoxycholic acid containing 1X Halt protease inhibitor cocktail (Pierce)). After centrifugation at 1000 g for 10 min to remove debris, 100 µL of caspase reagent containing the peptide substrate, benzyloxycarbonyl (Z)-DEVD-rhodamine R110, were added to the supernatant and the reaction was agitated in the dark at room temperature for 4 h. Fluorescence (excitation/emission: 496/520 nm) was measured in 96-well black plates (Corning Inc.) using a FLEXstation (Molecular Devices).

Adult worms exposed to statins were washed in PBS. Two worms were then ground up using a disposable plastic pestle in a 1.5 mL tube containing 100 µL lysis buffer and 1X Halt protease inhibitor cocktail. After centrifugation, 100 µL of caspase reagent were added to the supernatant and the reaction agitated in the dark at room temperature for 4 h. Fluorescence was measured as described above.

## Results

### Statins kill schistosomes in a concentration- and time-dependent manner

**Figure 1:**
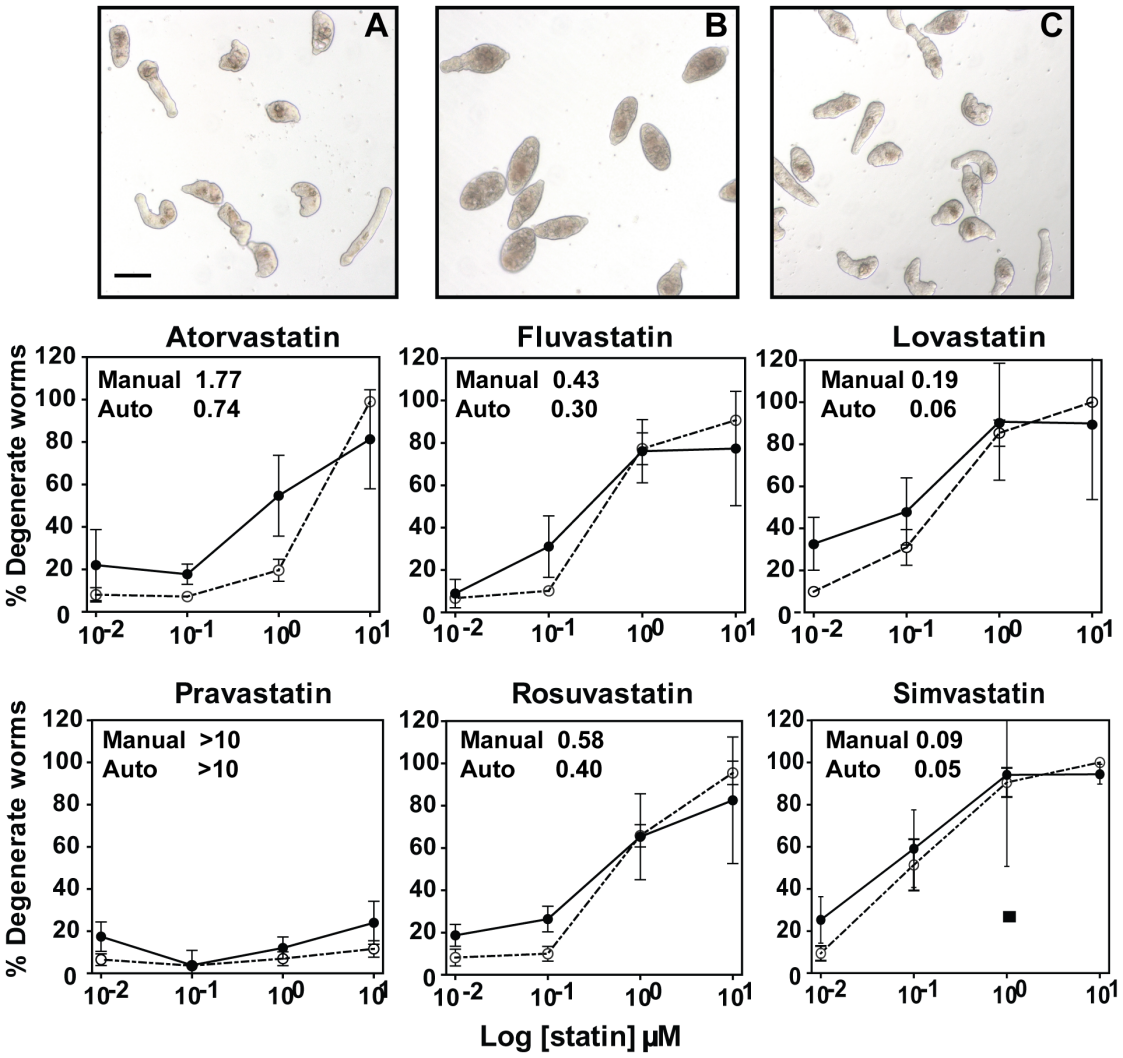
Concentration-dependent killing of *S. mansoni* somules by six commercial statins as measured manually and using a machine learning-based automated algorithm. Newly transformed somules were incubated out to four days in the presence of the concentrations of statins indicated. The percentage of degenerate worms was measured both manually (dashed curves) and using a new automated algorithm (solid curves) as described in the text. Points represent means ± S.D. across two independent experiments each in duplicate. ED_50_ values are inserted above the curves. Mevalonate (100 µM) prevented worm-kill by 1 µM simvastatin and is indicated by the single square point. Image panels display somules cultured for four days; (A) DMSO control, (B) 1 µM simvastatin (indicating the rounded and darkened (degenerate) response) and (C) 1 µM simvastatin plus 100 µM mevalonate. Bar in Panel A = 100 µm.

For six commercially available statins, dose-response curves, based on the appearance of somule degeneration, were prepared both manually and by an automated machine-learning approach (Figure 1). ED_50_ values were broad. Thus, for the automated method at Day 4, values ranged from 0.05 and 0.06 µM for simvastatin and lovastatin, respectively, to >10 µM for pravastatin. The process of degeneration was consistent among the statins whereby the normally translucent and flexible parasite (Figure 1 panel A) became darkened and ovoid with decreased movement (Figure 1, panel B). Death was invariably the consequence of continued incubation. The dose-response data calculated using the automated method for the six statin compounds correlated significantly with those generated manually: Table 1 lists the Pearson product-moment correlation coefficients obtained between the two sets of curves, as well as the significance (*p*-value) of the correlation determined by Student's *t*-test.

**Table 1:** Cidal activity of statins against somules *in vitro*: dose-response correlations between the automated and manual assays.

	Atorvastatin	Fluvastatin	Lovastatin	Pravastatin	Rosuvastatin	Simvastatin
Correlation	0.864	0.959	0.991	0.948	0.987	0.983
*p*-value	0.136	0.0410	8.985E-3	0.0523	0.0130	0.0171

*P*-values were determined using Student's *t*-test.

Degeneration of somules was also time-dependent. For example, with simvastatin, degeneracy appeared by Day 2 of the incubation at the greatest concentration of 10 µM and was essentially complete (i.e., dead parasites) by Day 4 at both 10 and 1 µM (Figure S1). Parasite degeneration by simvastatin (Figure 1 panel C and lower right panel) or lovastatin (not shown) was preventable by incubation with excess (100 µM) mevalonate, the product of HMGR. Thus, SmHMGR is the relevant statin target. Rescue by mevalonate was concentration-dependent with a minimum of 100–200 µM being required for maximal protection of the parasite (Figure S2).

**Figure 2:**
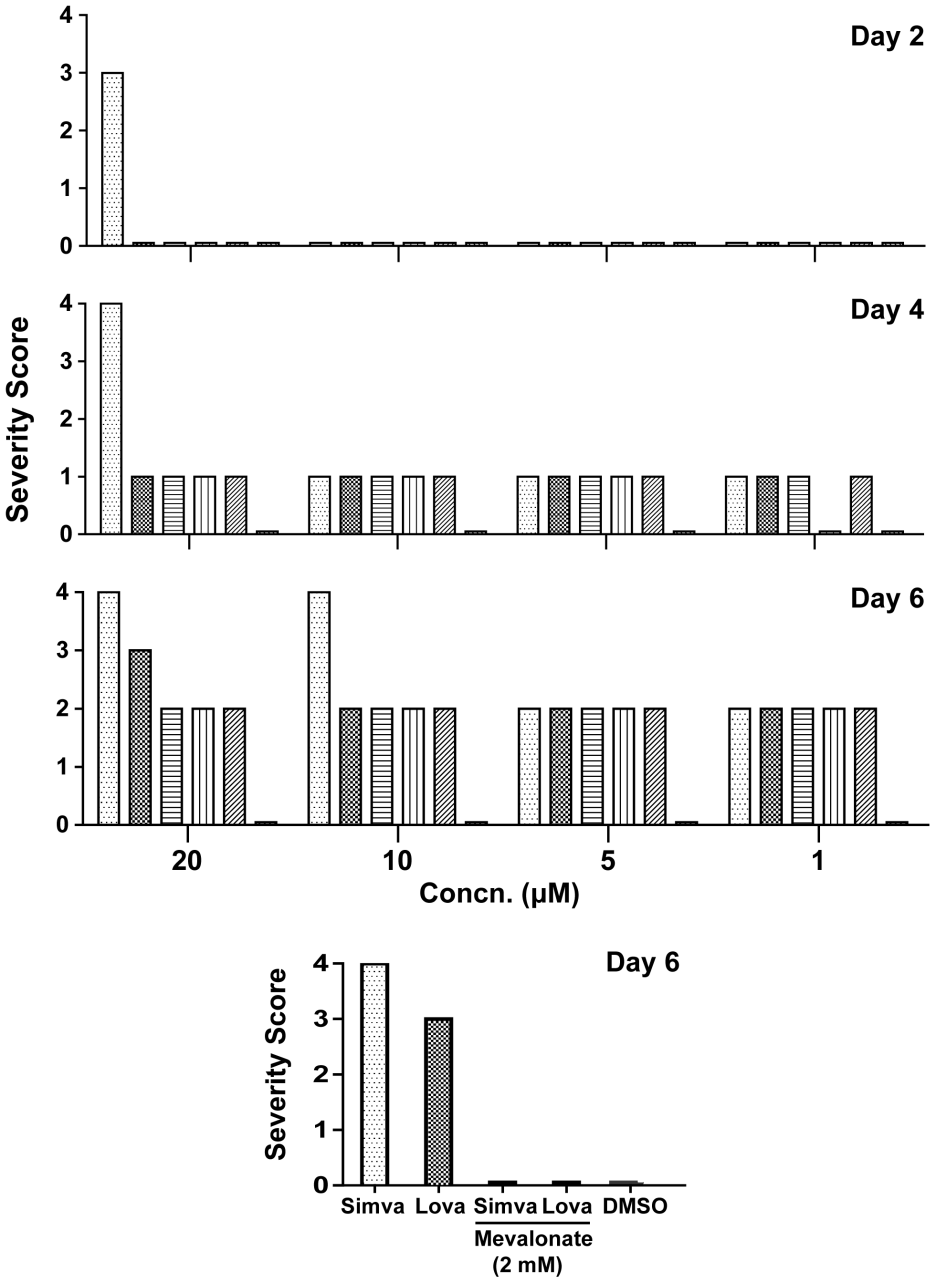
Time- and concentration-dependent effects of six statin drugs on adult *S. mansoni in vitro*. Adult worms were incubated out to six days in the presence of the concentrations of statins indicated. A severity score was devised based on previously employed phenotype descriptors that record changes in movement, shape, translucence, surface integrity, and the ability of the parasite to adhere to the floor of the culture plate well [35]. Each descriptor was assigned a score of 1, except where damage to the parasite's surface was evident, in which case the maximum score of 4 was awarded. For each concentration tested, each bar (from left to right) represents simvastatin, lovastatin, atorvastatin, fluvastatin, rosuvastatin and pravastatin. At the bottom, co-incubation with mevalonate (2 mM) for three days prior to the addition of 20 µM simvastatin or lovastatin prevented worm degeneration. Representative data from two independent experiments are shown.

Likewise for adult *S. mansoni*, simvastatin was the most potent drug whereas pravastatin was without effect (Figure 2). Thus, by Day 2 in the presence of 20 µM simvastatin, worms had become slower and darker, and did not adhere to the culture well floor (severity score of 3): the other statins were without apparent effect. By Day 4, simvastatin at 20 µM had shrunk and immobilized the worms, and the parasite tegument had been disrupted (a maximum severity score of 4). By Day 6, the worms were dead. From Day 4 through Day 6, the other statins caused some darkening and slowing of the worms, although, with the exception of lovastatin a severity score of 2 was never exceeded. Incubation of worms for three days with 2 mM mevalonate prior to the addition of 20 µM simvastatin and lovastatin prevented worm degeneration (Figure 2).

### RNAi of HMGR is specific to the target

**Figure 3:**
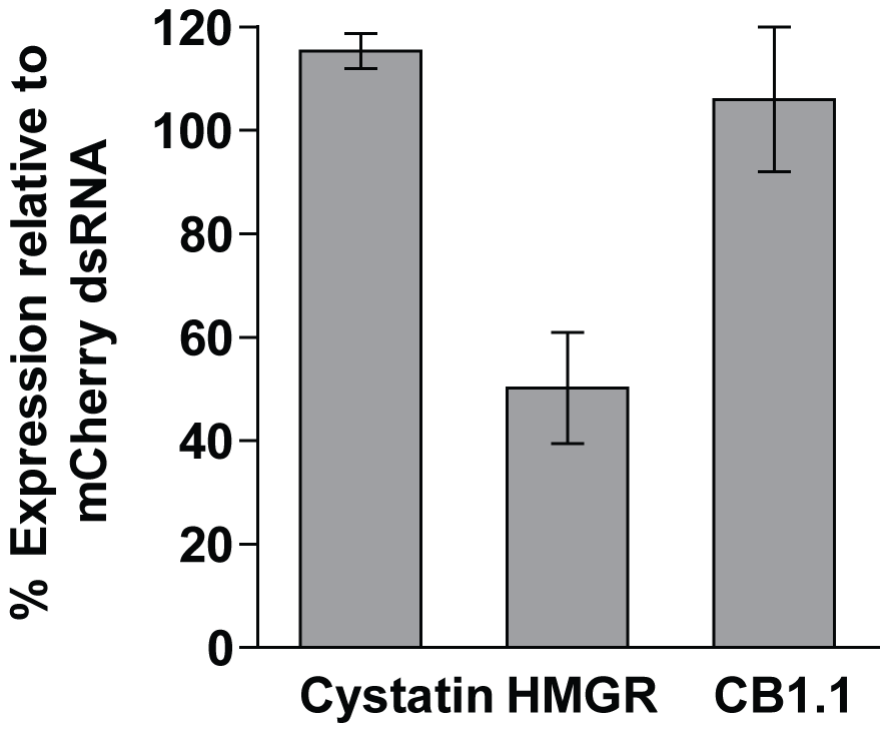
RNAi of SmHMGR is selective for the target. Newly transformed somules were incubated with HMGR- or mCherry-dsRNA for seven days as described in the text. RNAi was measured by qRT-PCR and data expressed relative to data for mCherry dsRNA controls. To assess for potential off-targeting by HMGR-dsRNA, the expression of a *S. mansoni* cysteine protease inhibitor, cystatin (Cys: AY334553.1), and the gut-associated cysteine protease, cathepsin B1.1 (AJ506157), was also measured. Bars represent means ± S.D. across two independent experiments each in duplicate. Using Student's *t*-test, gene suppression in the HMGR-dsRNA treated sample is significantly different from the mCherry controls (*p*<0.01).

The above data suggest that HMGR is the relevant molecular target for the cidal activity of statins. To confirm this, we applied transient RNAi as a reverse genetics strategy to target HMGR transcription. In advance, however, we formally demonstrate that HMGR is expressed in somules and other developmental stages (Figure S3). Incubating somules for seven days in the presence of 30 µg/mL HMGR dsRNA (added every third day) decreased HMGR transcript levels by 50% (Figure 3). RNAi of HMGR was apparently specific as no off-target modulation of *S. mansoni* cystatin or cathepsin B1.1 transcript levels was noted, findings that are consistent with our previous data using transient RNAi in somules [37].

### RNAi of HMGR *in vitro* kills somules, an outcome that can be prevented by mevalonate

**Figure 4:**
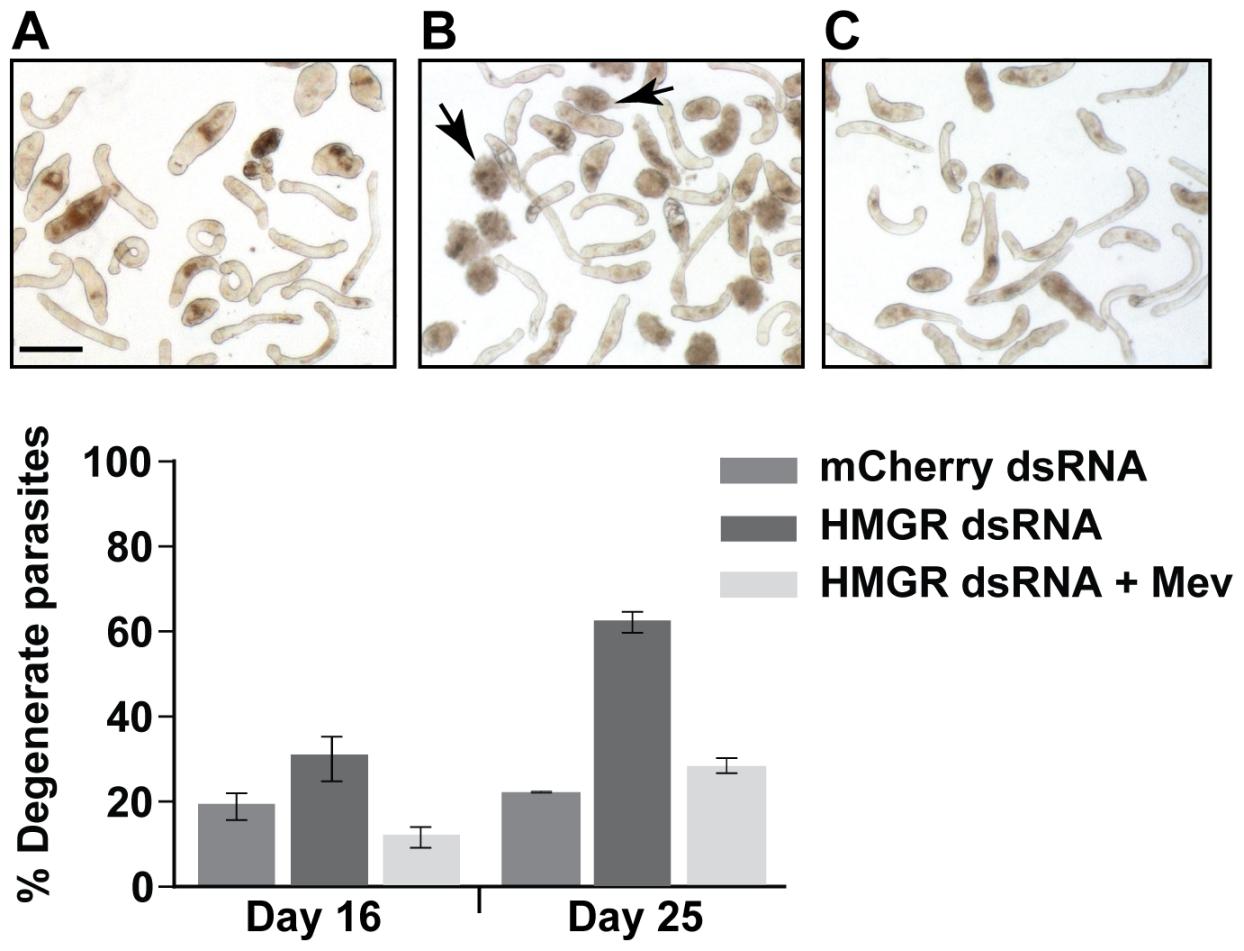
RNAi of SmHMGR kills somules and killing is preventable by excess mevalonate. Newly transformed somules were incubated for the times indicated in the presence of mCherry-dsRNA (control) or HMGR-dsRNA with and without the presence of 500 µM mevalonate, as described in the text. Bars represent means ± S.D. across two independent experiments each in duplicate. The number of degenerate worms in the HMGR-dsRNA-treated samples taken on Day 25 is significantly different from both the mCherry controls and HMG-dsRNA-treated samples that had also been incubated in the presence of mevalonate (Student's *t*-test; *p*<0.01). Image panels display somules on Day 25 incubated with (A) mCherry-dsRNA (control), (B) HMG-dsRNA and (C) HMG-dsRNA plus 500 µM mevalonate. Arrowheads highlight a dead (left) and degenerate worm (right). Bar in panel A = 100 µm.

No phenotype was evident by the seventh day of incubation with HMGR dsRNA when somules were harvested for qRT-PCR analysis. Therefore, we extended the incubation time in the hope that an obvious phenotype might appear and in the knowledge that HMGR transcripts were only decreased by 50%. We continued to add 30 µg/mL HMGR dsRNA and exchange the culture medium every three days. By day 16, a small but non-significant (relative to mCherry controls) number of the parasites incubated with HMGR dsRNA had become rounded, darkened and less motile (Figure 4). The progress of degeneration continued through the end of the experiment on day 25 by which time 62% of the parasites had degenerated relative to the 21% background recorded for parasites in the presence of mCherry dsRNA. Importantly, RNAi-induced degeneration could be prevented by including mevalonate (500 µM) in the incubation, thus identifying HMGR as the relevant target.

### RNAi of HMGR *in vitro* limits subsequent parasite survival *in vivo*

**Figure 5:**
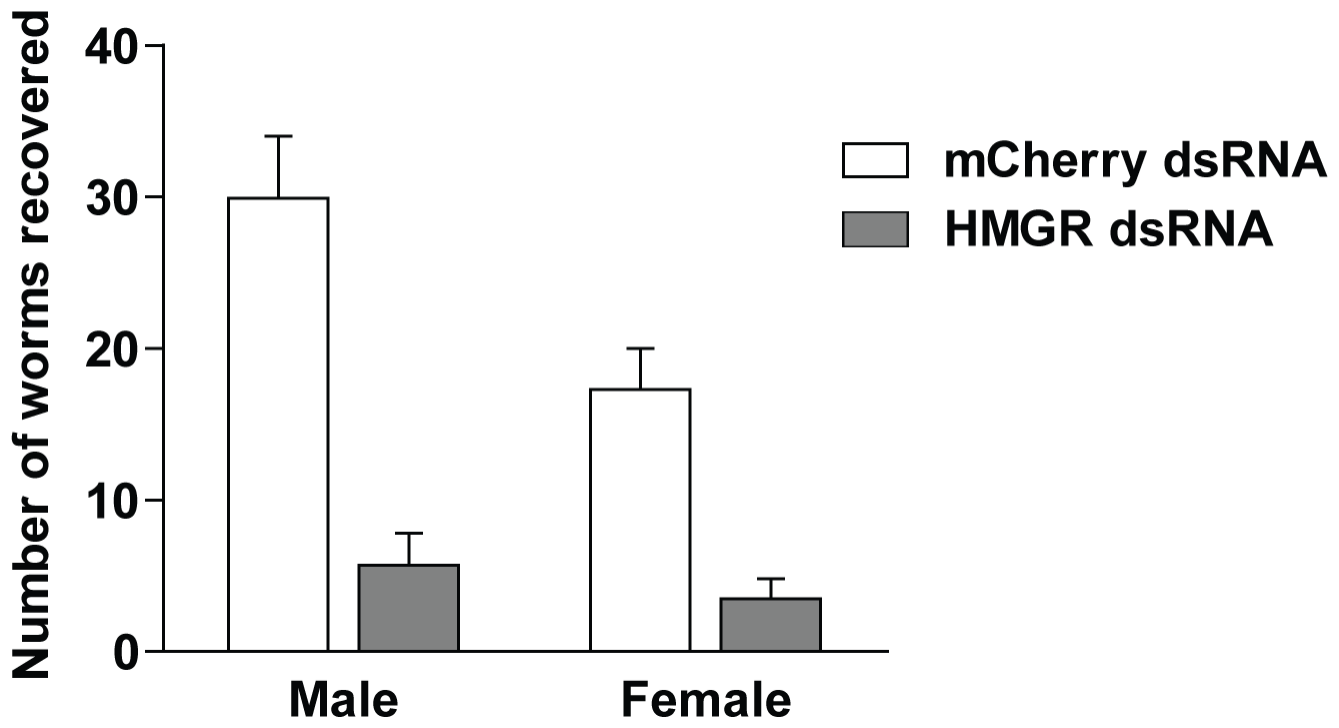
RNAi of SmHMGR severely compromises parasite survival *in vivo*. Newly transformed somules were incubated for 2.5 days in the presence of mCherry dsRNA or HMGR dsRNA as described in the text. Parasites were then injected subcutaneously into Swiss Webster mice (5 mice per dsRNA treatment). After 34 days, mice were euthanized and the portal system perfused to recover parasites for counting. Bars represent means ± S.D. across two independent experiments. Using Student's *t*-test, worm recovery after exposure to HMGR-dsRNA was significantly different from mCherry controls (*p*<0.01).

We asked whether somules exposed *in vitro* to HMGR dsRNA would thrive when transferred into mice, which are typically used as an animal model of schistosome infection. Somules were incubated in the presence of mCherry or HMGR dsRNA that had been added twice daily at 60 µg/ml for 2.5 days and then injected sub-cutaneously into mice. Mice were sacrificed 34 days later and the hepatic portal system perfused to recover parasites. Irrespective of the dsRNA employed, all parasites recovered appeared normal in size, shape, motility and translucence (not shown). However, relative to mCherry dsRNA controls, the number of worms exposed to HMGR dsRNA was decreased by 81 and 80% for males and females, respectively (Figure 5).

### Chemical and reverse genetic targeting of HMGR induces apoptosis in somules

**Figure 6:**
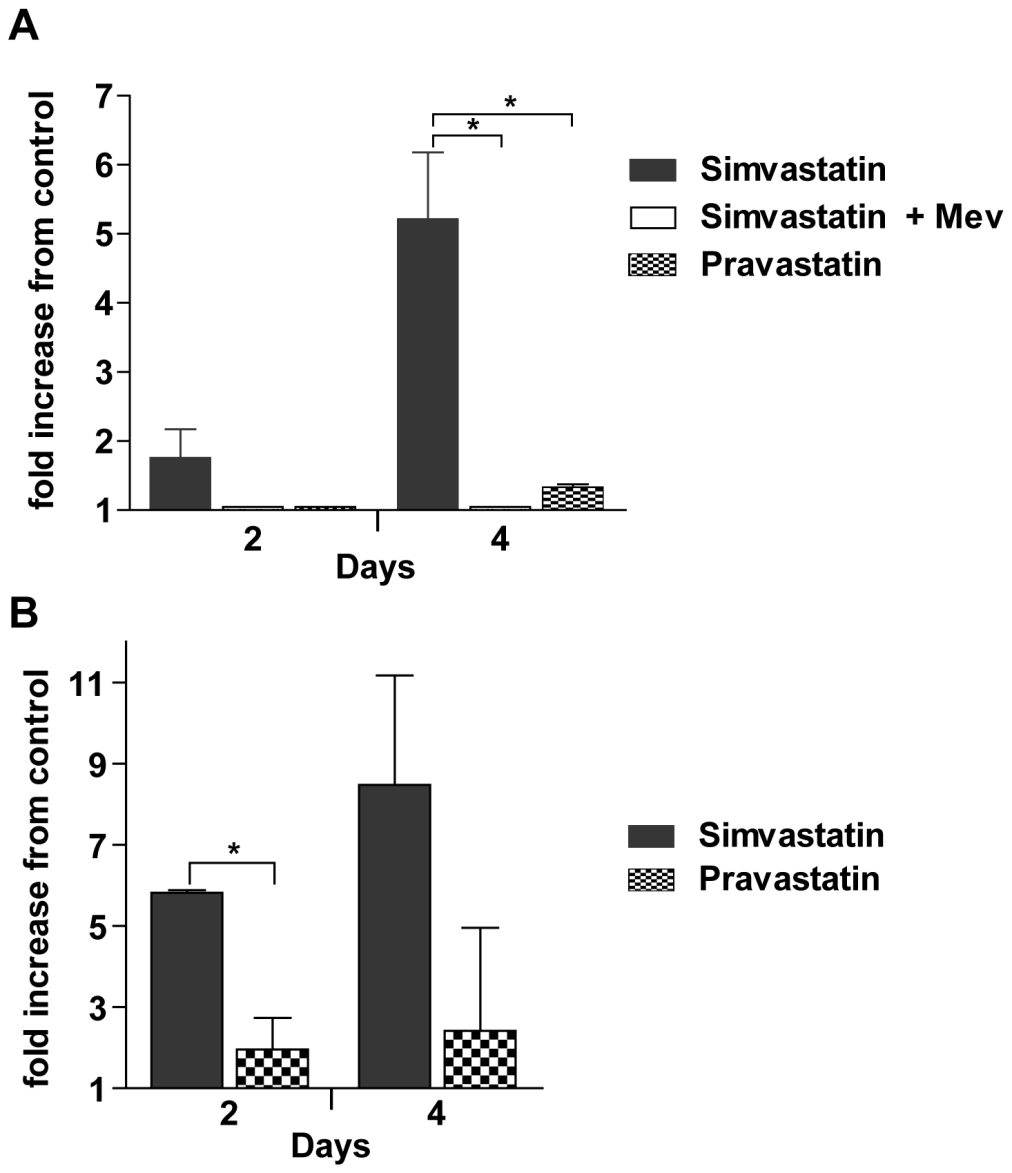
Simvastatin, but not pravastatin, induces apoptosis in somules and adult worms. **A**. Newly transformed somules were incubated in the presence of either 10 µM simvastatin (±100 µM mevalonate) or pravastatin for the times indicated. Caspase activity was then measured using the fluorescent substrate Z-DEVD-R110 as described in the text and is expressed relative to DMSO controls. Bars represent means ± S.D. across two independent experiments. Significance (p<0.05) was measured using Student's *t*-test and is indicated with an asterisk. **B**. As for A, but employing adult worms. Bars represent means ± S.D. across two independent experiments. Significance (p<0.05) was measured using Student's *t*-test and is indicated with an asterisk.

To understand the mechanism associated with the lethality of statins to *S. mansoni* somules and adults, we assayed for the activation of caspases as an indicator of apoptosis, which is a common outcome upon inhibition of HMGR in cancer cells (see Discussion). Somules incubated for two days in the presence of 10 µM simvastatin showed a small but non-significant increase in caspase activity compared to DMSO controls (Figure 6A). By Day 4, however, the activity was five-fold that of control. For both time points, mevalonate at 100 µM prevented the activation of caspase activity and the non-cidal statin, pravastatin (10 µM) was without effect.

Adult parasites that had been incubated for two days with 10 µM simvastatin showed a six-fold increase in caspase activity compared to DMSO controls (Figure 6B). By Day 4, activity was a non-significant eight-fold that of control. For both time points, the non-cidal statin, pravastatin (10 µM), did not significantly activate caspase activity over DMSO controls.

**Figure 7:**
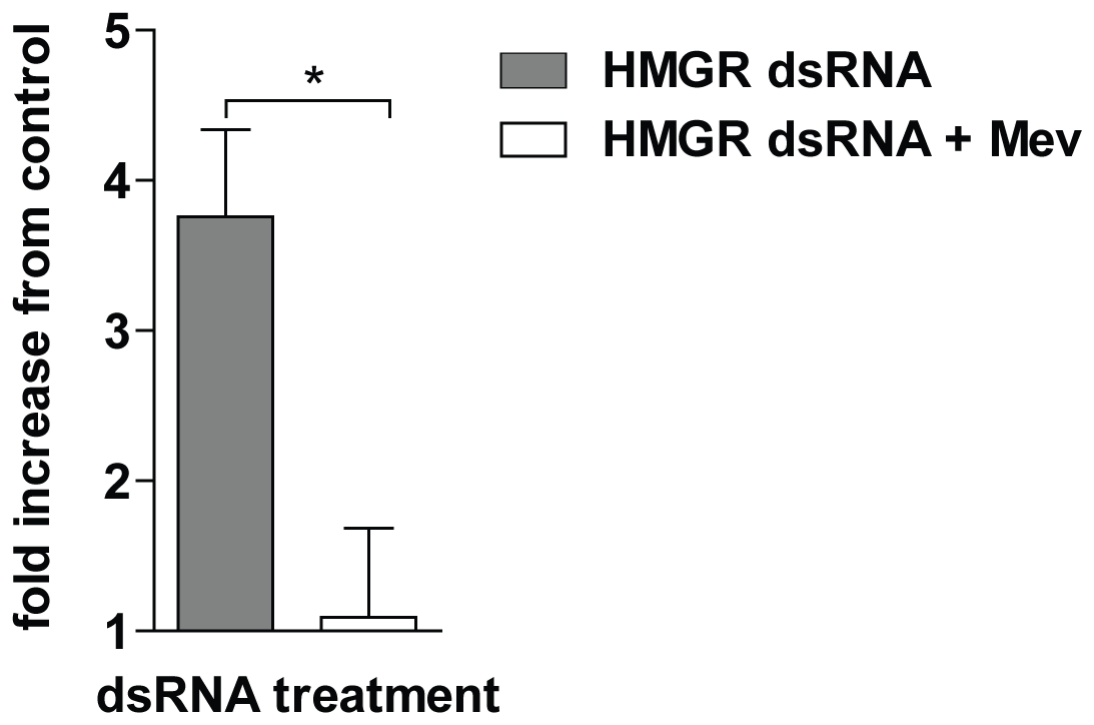
RNAi of SmHMGR induces apoptosis in somules, which is preventable by excess mevalonate. Somules were incubated with dsRNA to HMGR (±500 µM mevalonate) or mCherry for 25 days. Caspase activity was then measured using the fluorescent substrate Z-DEVD-R110 as described in the text. Data are expressed as the fold increase in caspase activity relative to somules exposed to mCherry dsRNA. Bars represent means ± S.D. across two independent experiments. Significance (p<0.05) was measured using Student's *t*-test and is indicated with an asterisk.

We also assayed for activation of caspases in somules exposed to HMGR RNAi. Somules were incubated in the presence of HMGR or mCherry dsRNA as described above. On day 25, caspase activity was measured in supernatants of lysed worms. Compared to mCherry-exposed controls, RNAi of HMGR induced a four-fold increase in caspase activity: mevalonate at 500 µM prevented caspase activation (Figure 7).

## Discussion

Here, we demonstrate that statin drugs, which are now ubiquitously employed to treat hypercholesterolemia, kill *S. mansoni* somules and adults *in vitro*. Statins target human HMGR, the rate-limiting step in the mevalonate pathway that is responsible for biosynthesis of a number of key products required for cell growth and survival, including cholesterol, farnesyl pyrophosphate and geranyl pyrophosphate [4], [56]–[59]. We show that the SmHMGR ortholog is the relevant target for the schistosomicidal activity of statins as worm-kill is prevented by excess mevalonate, the product of HMGR. RNAi of HMGR kills the parasite and, again, lethality is prevented by providing excess mevalonate. Finally, we demonstrate that HMGR is crucial to the survival of somules upon their transfer to mice post-RNAi of HMGR *in vitro*. Parasite death, either via statins or specific RNAi of HMGR, is associated with apoptosis. Together, our chemical and reverse genetics data demonstrate that *S. mansoni* HMGR is an essential gene and the relevant target of statin drugs which may form the basis for a new anti-schistosomal drug therapy.

In terms of the methodologies employed to quantify the cidal activity of statins against somules, the data generated manually were cross-validated with a novel automated approach that involves biological imaging and machine learning using support vector machines. The approach, novel in its application to drug screening for schistosomiasis, can monitor phenotypic changes in multiple parasites simultaneously and provides for determination of complete dose- and time-response curves, including ED50 values. The description of the automated machine learning-based approach along with experiments on a larger dataset of statin and non-statin drugs will be the subject of a separate report.

Our decision to investigate statins and SmHMGR as a possible therapeutic axis for schistosomiasis was triggered by our previous phenotypic (whole-organism) screen of a small molecule collection that included drugs approved for use with humans [35]. Specifically, statins emerged as one of several drug classes that consistently damaged and killed somules after seven days at 1 µM. Our subsequent search of the literature for precedent regarding statins, HMGR and schistosomes identified *in vivo* [60] and *in silico* [61] studies that further suggested their relevance. Finally, of a number of reports by Bennett and colleagues [32], [34], perhaps the most pertinent to this discussion was the demonstration of near cure of adult and juvenile *S. mansoni* infections in mice that had been provided *ad libitum* with lovastatin (a.k.a. mevinolin) mixed into mouse chow [34]. Thus, our present data are consistent with and expand upon those from other sources, and encourage the continued exploration of statins and the SmHMGR target to develop a new chemotherapy for schistosomiasis (see below).

We determined that statins and/or RNAi of HMGR induce apoptotic caspase activity in schistosomes as measured with peptidyl (DEVD) caspase substrates. Caspases-3 and −7 (ACU88129 and ACU88130, respectively) are expressed in *S. mansoni* [55] and the existence of an apoptosis pathway in *S. mansoni* [62] and *S. japonicum* has been described [63]. Apoptosis is a typical outcome for many cancer cell lines exposed to statins *in vitro* whereby blockading of what is often a dysregulated mevalonate pathway [64] leads to a myriad of downstream effects. Indeed, the abundant pre-clinical evidence for statins as potential anti-cancer agents has been translated to over 100 clinical trials currently registered at the ClinicalTrials.gov website (operated by the United States National Institutes of Health; queried on October 10^th^ 2013 with the terms ‘statin’ AND ‘cancer’) to test statins either as a monotherapy or in combination with other agents. Among the defects caused by statins is the defective prenylation of small GTPases (e.g., Ras, Rho and Rac) that are necessary for correct cell division [13], [14], [65]. In schistosomes, small GTPases belonging to the Ras superfamily that regulate development are farnesylated. Specifically, RhoA, also known as Rho1, which is geranyl-geranylated, may contribute to regulating the dynamics of the actin cytoskeleton [66]–[68].

Interestingly, of the statins known to induce apoptosis in cancer cells in culture, pravastatin stands out as being generally the least effective [14]. Pravastatin is the most hydrophilic of the statins in use and is not readily taken up by cancer cells unlike the other more lipophilic drugs. In like fashion, we noted pravastatin's inability to kill both adults and somules, and induce caspases. Indeed, the statin ED_50_ values against somules *in vitro* trended with lipophilicity whereby the lipophilic statins (simvastatin>lovastatin ≈ atorvastatin ≈ fluvastatin> rosuvastatin) were generally more active than the hydrophilic pravastatin [69]. Likewise for adult worms, simvastatin and pravastatin were consistently the most and least active of the drugs tested, respectively.

**Figure 8:**
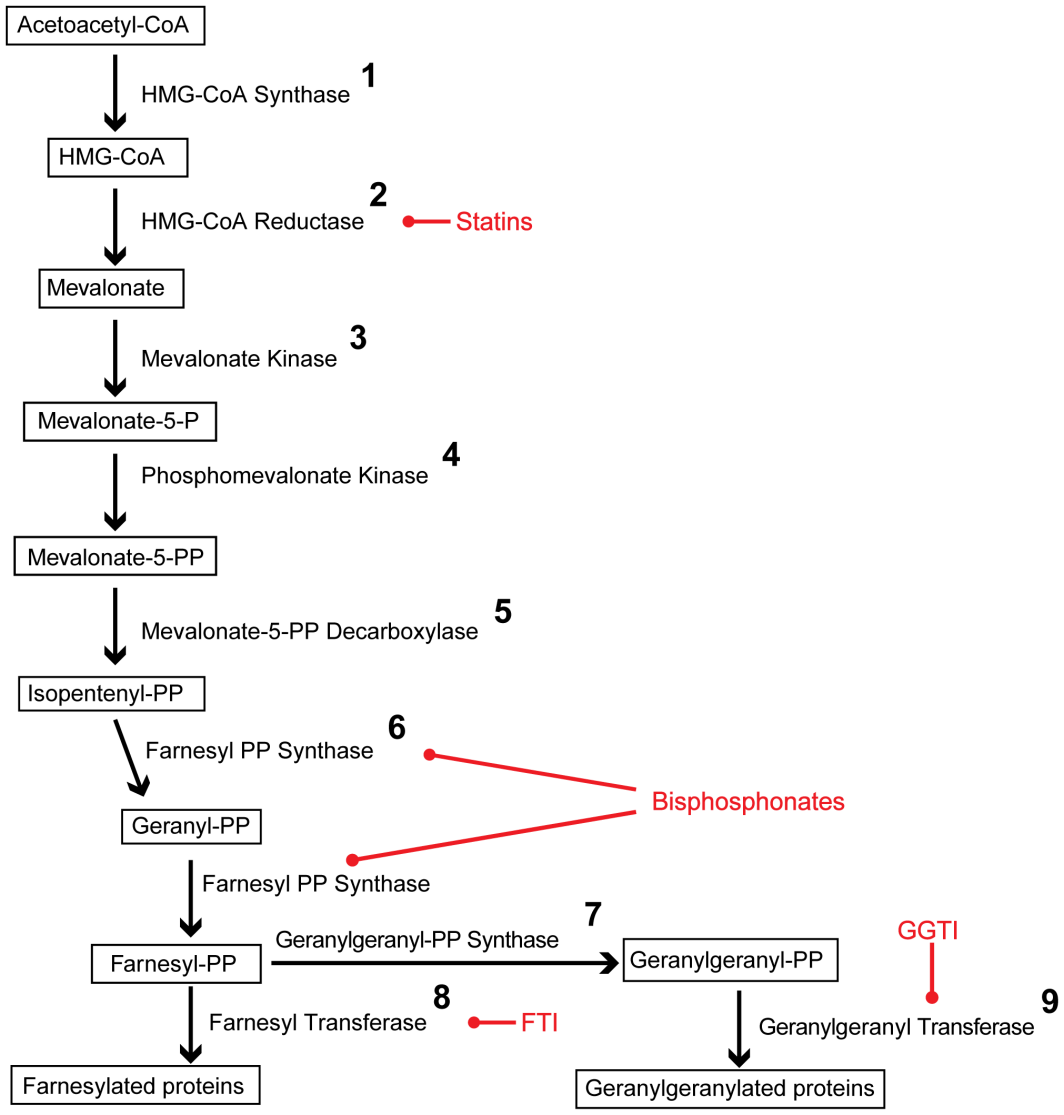
Overview of the mevalonate pathway in *S. mansoni*. The enzymes are numbered in bold typeface and their respective products are boxed; synthetic inhibitors are indicated in red. GenBank entries for each enzyme are: **1**- CCD58635; **2**- CAZ30720; **3**- CCD79354; **4**- CCD76423; **5**- CCD60126; **6**- CCD78373; **7**- CCD81269; **8**- α-subunit- XP_002580188; β-subunit- CCD82339; **9**- α-subunit- CCD77887; β-subunit- CCD79051. FTI  =  farnesyl transferase inhibitor; GGTI  =  geranylgeranyl transferase inhibitor.

As judged by manual query of the *S. mansoni* genome (GeneDB, Version 4.0), a complete mevalonate pathway is encoded by the parasite (Figure 8). In addition to SmHMGR, there are a number of other enzymes downstream in the pathway that could be interrogated for development of an anti-parasite therapy based on clinical precedent in oncology (farnesyl transferase [70]–[73]) and osteoporosis (farnesyl diphosphate synthase [4], [74], [75]). Indeed, these interrogation points are also under evaluation for treatment of other parasitic diseases. Thus, bisphosphonate inhibitors of farnesyl diphosphate synthase have been investigated as anti-trypanosomal agents [76], [77] and farnesyl transferase inhibitors inhibit the growth of *Plasmodium falciparum, Leishmania mayor, Trypanosoma cruzi, Giardia lamblia* and *Entamoeba histolytica* [18], [78].

In light of the specific requirement for short course (preferably single dose) oral therapy of helminth diseases (incl. schistosomiasis) of humans [24], [79], [80], the current data for statins suggests that we are still short of an acceptable efficacy profile. First, using lovastatin, Chen et al. [34] could only demonstrate clearance of *S. mansoni* from the mouse infection model with an extended dosing regimen of 14 – 17 days and using high concentrations of lovastatin (estimated at 640 mg/kg/day provide *ad libitum* in mouse chow). By contrast, a single, oral, 500 mg/kg dose of the current anti-schistosomal drug, PZQ, kills >90% of adult worms in mice [81]. Second, in the present *in vitro* work, whereas > 60% of somules had degenerated by the fourth day of incubation in the presence of 1 µM of most statins, adult parasites were more resilient, i.e., only simvastatin at 20 µM caused severe damage on Day 4, whereas the other five statins had a relatively minor impact. This concentration is well above the approximately 0.1 µM maximal plasma levels of statins used to treat hypercholesterolemia based on a once daily chronic dosing regimen [82]. Encouragingly, however, no dose limiting toxicity has been noted in humans up to plasma levels of 12.3 µM lovastatin when administered every six hours over four days [83]. In fact, maximum plasma levels were achieved within 24 h [83] suggesting that, under a relatively acute dosing regimen, plasma levels of statins that are safe yet parasiticidal may be achievable. Moving beyond the commercially available statins, there is also likely considerable scope for improving selectivity and inhibition kinetics toward the schistosome HMGR in a formal drug development program employing a statin library and *de novo* chemical synthesis. In support of a program to optimize a ‘schistostatin’ is the approximately 50% primary sequence difference in the catalytic domains of the human (SQ1L_A) and SmHMGR (AAA29896.1) orthologs that presumably contributed to the demonstration of differences in inhibition kinetics between the enzymes using a small series of statin analogs [29].

A relevant consideration for developing statins as anti-schistosomals is their cidal activity against immature parasites, both *in vitro* (see above) and in a mouse infection model [34], which stands in contrast to PZQ's well-known poorer efficacy against the younger developmental stages [81], [84]. Not least, the possibility of a combination therapy with PZQ is worth investigating in one effort to preserve the clinical utility of the latter drug given the current momentum to expand its deployment as a monotherapy and the associated increased risk of resistance [24], [36]. Finally, given the evidence surrounding HMGR and statins in schistosomes and the conservation of HMGR and the mevalonate pathway among helminths, statins may have potential as general anthelmintics; either as the preferred short-course therapy, or, for example with filariasis, for which longer drug administration regimens are under consideration [85], [86].

## Supporting Information

Figure S1 **Time-dependent killing of** ***S. mansoni*** **somules by simvastatin** ***in vitro*** **.** Newly transformed somules were cultured out to four days in the presence of simvastatin at the concentrations indicated. Time point data represent means ± S.D. across two independent experiments each in duplicate.
(TIF)

Figure S2 **The prevention of statin-induced somule death by simvastatin using mevalonate is concentration-dependent.** Newly transformed somules were cultured for four days in the presence of simvastatin at 1 µM and the concentrations of mevalonate indicated. Data are displayed as means ± S.D. across two independent experiments each in duplicate.
(TIF)

Figure S3 **Sequences of primers employed for dsRNA synthesis and qRT-PCR.** ***S. mansoni*** **developmental stages.** Data are displayed as means ± S.D. across two independent experiments each in duplicate. Transcript expression levels are normalized to that of cytochrome C oxidase I (GenBank accession number, AF216698) [52], [53] and the data are displayed according to [54]. NTS  =  newly transformed somules.
(TIF)

Table S1 **Sequences of primers employed for dsRNA synthesis and qRT-PCR.**
(XLSX)
