# Supplementary figures and images for: Chemical and Genetic Validation of the Statin Drug Target to Treat the Helminth Disease, Schistosomiasis
Source: PLoS One. 2014 Jan 29;9(1):e87594. doi: 10.1371/journal.pone.0087594 (PMC3906178; doi:10.1371/journal.pone.0087594)

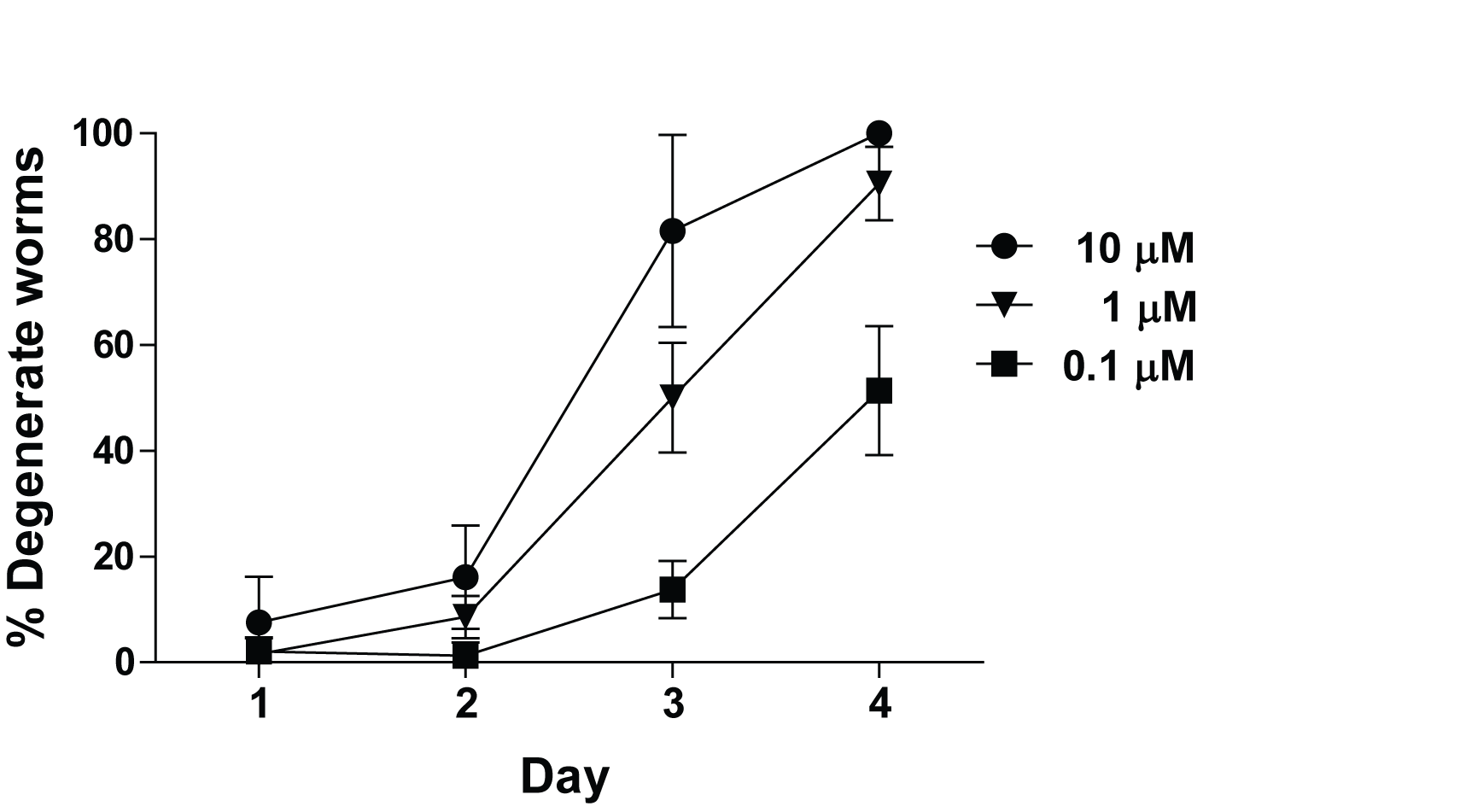

Supplement: Figure S1 — Time-dependent killing of S. mansoni somules by simvastatin in vitro . Newly transformed somules were cultured out to four days in the presence of simvastatin at the concentrations indicated. Time point data represent means ± S.D. across two independent experiments each in duplicate. (TIF) [file pone.0087594.s001.tif]

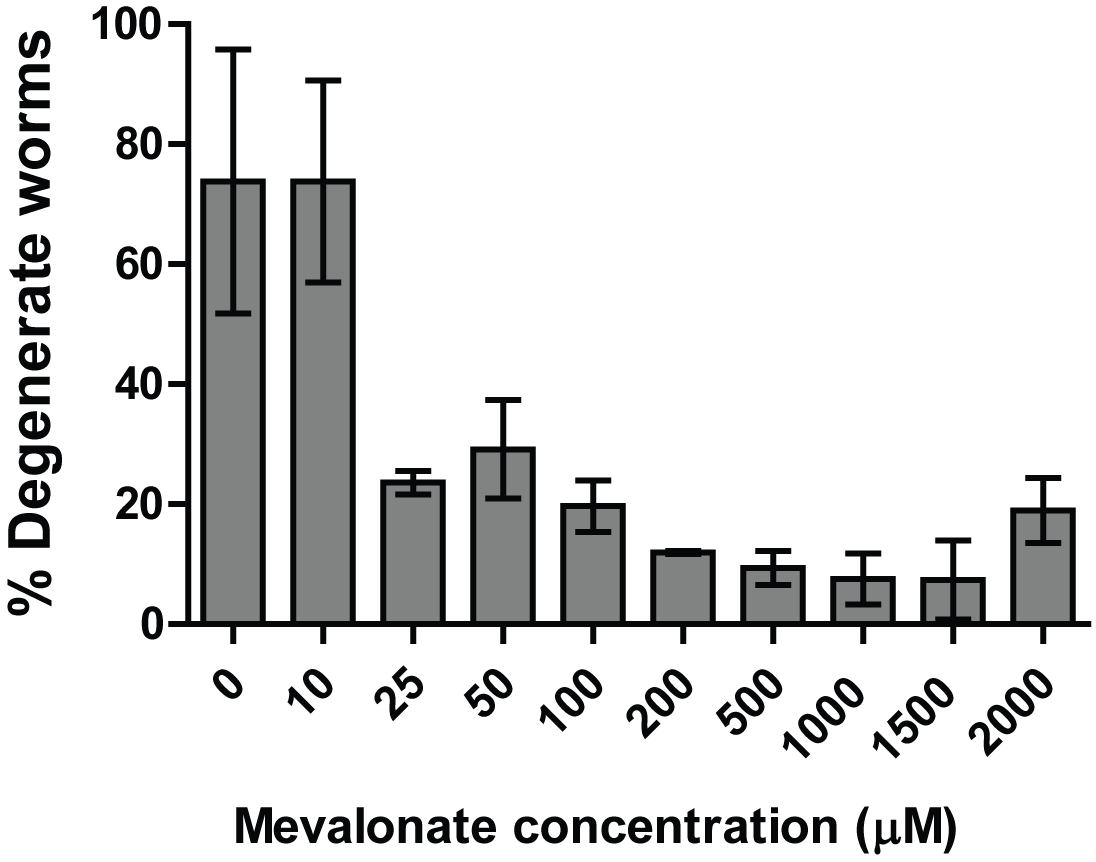

Supplement: Figure S2 — The prevention of statin-induced somule death by simvastatin using mevalonate is concentration-dependent. Newly transformed somules were cultured for four days in the presence of simvastatin at 1 µM and the concentrations of mevalonate indicated. Data are displayed as means ± S.D. across two independent experiments each in duplicate. (TIF) [file pone.0087594.s002.tif]

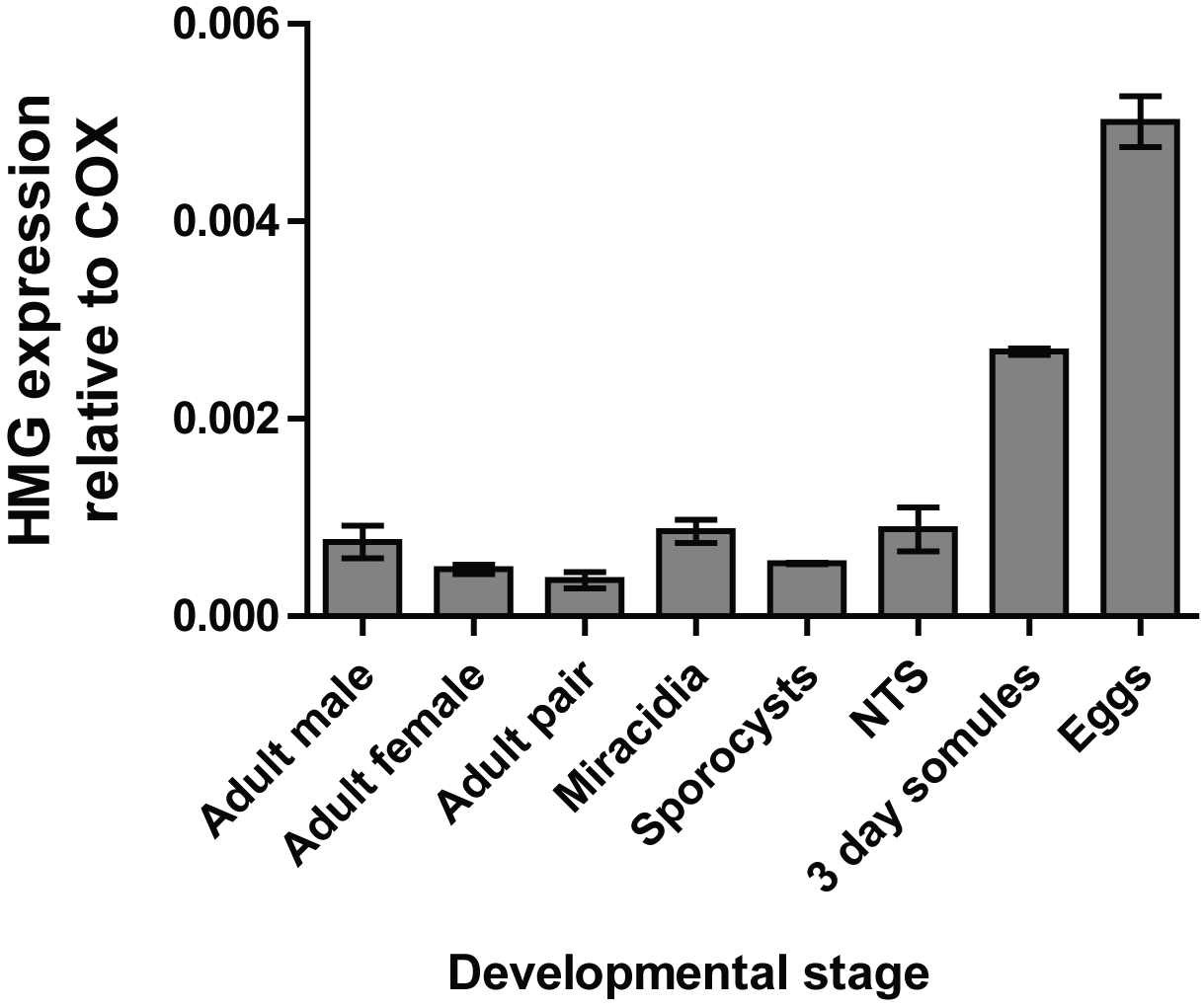

Supplement: Figure S3 — Sequences of primers employed for dsRNA synthesis and qRT-PCR. S. mansoni developmental stages. Data are displayed as means ± S.D. across two independent experiments each in duplicate. Transcript expression levels are normalized to that of cytochrome C oxidase I (GenBank accession number, AF216698) [52], [53] and the data are displayed according to [54]. NTS = newly transformed somules. (TIF) [file pone.0087594.s003.tif]
